# Supplementary material for: Assessment and site-specific manipulation of DNA (hydroxy-)methylation during mouse corticogenesis
Source: Life Sci Alliance. 2019 Feb 27;2(2):e201900331. doi: 10.26508/lsa.201900331 (PMC6394126; doi:10.26508/lsa.201900331)
Supplement: Supplementary file 3 [file LSA-2019-00331_TableS1.docx]

Table S1. Primers and gRNAs used in this study

| name | forward primer | reverse primer | Tm |
| --- | --- | --- | --- |
| BS_chr11:80480572-80480726 | TTTTTTATTTTTGTTGTTTTTATAGTGGTA | AAATCCAATCCTAAACTATACTCCTCC | 58°C |
| BS_chr2:35628200-35628427 | TTTTGGAGGTGAAGTTTGATATGA | ATCTAAAACAAATCCAAATTACCAATTT | 58°C |
| BS_chr10:121029488-121029712 | TGGAAGATAAGTTATGTAAGGTTTTT | TTCTTTTATACTTCTATCAATCTATCCTC | 55°C |
| BS_chr13:97246233-97246498 | TGATTTTATAATTAATAAATGGATTATGGT | AACAAAAAACTCTATATCTCCACCC | 58°C |
| BS_chr11:98333236-98333393 | AATTAGGGGGAAATTGGAATAGAG | CCTCTAAAAAACCTAAAAAAAACCAA | 58°C |
| BS_chr1:190161836-190162108 | GGGTTGTTTTTTGAAGAGTTGTTATATATA | CCCCAAAATTCAAAACAAAATATTA | 58°C |
| BS_chr16:89973866-89974252 | AAGGTTAGGTATTTGGGAATTTTTG | TACACAAACAAAAACAAATCTCCAC | 62°C |
| BS_chr6:125164858-125165171 | GGAAGTTTAAGTTTGGGAGATGATG | CCCAAAAAAACCTCAAAATCAAC | 62°C |
| BS_chr7:105777815-105778184 | TTGTTTATTTTATGTGGATTGATATAGTAGGT | CAAAAATAAAATAAATAAAACAAACCTA | 62°C |
| BS_chr7:105778370-105778731 | GTAAGATTGTTTATTGGGGTTTGAAA | CTTATTCTTCCACTTCTAACTCAACTAC | 62°C |
| offtarget#1_chr8:5994340-5994632 | TTGAGGGGTGATTTTGATAAAG | ACCACTCAAACAAAACAAACACTTTTA | 62°C |
| offtarget#2_chr11:85277228-85277728 | AGTGGAAAGATATAGTAGGTTTTGGG | CCAAACAAAAATAACAAATCACCCTA | 62°C |
| offtarget#3_chr4:10384632-10385132 | GTGTTAGTGTTTAGTTAGAATTTTGGGT | CTATCCCAAAATAACATATAAACTTCAC | 62°C |
| offtarget#4_chr3:65654161-65654661 | GGTTGGGATATAAAGTTAAAGAATTG | TAAAAATTACTAAAATCATTTCCCCAAA | 62°C |
| offtarget#5_chr1:36503230-36503426 | GTGGAATGAGATAGTTGGTTTTTGAAAG | CATCAAATCCCATTACAAATAATTAAATAC | 62°C |
| offtarget#6_chr8:99125976-99126211 | GTTGATAGATGATAGATATATTTTTTGTAGAG | CAAACACATTAAAATAAACCCTAAATTTAAC | 62°C |
| offtarget#7_chr4:139611562-139612002 | TTTTAGATTAAGGTTGAGAGGAGAA | CTCACTCAACAAAAATATAAATTAAAAACC | 62°C |
| offtarget#8_chr14:98847763-98847942 | TTTTGGGTATAATTTTGTTTGTTTTTTG | CACACCTAAATAATACCTATATAATATTTC | 62°C |
| offtarget#9_chr15:101550328-101550624 | GGGGTGATGTAGGTAGAAGATTTATAGG | CCACCTCTACCTCCCAAAATCATATC | 62°C |
| offtarget#10_chr13:62889156-62889301 | GGTTAGTTAGTTAGGTTAGTGTAATTAG | CTCCTTTCTTTCCTAATATCTATATAAC | 62°C |
| amplification of Tet1 and dTet1 | AAAACGCGTGCTGCACCCTGTGACTGTGATG | AAATCTAGATTAGACCCAACGATTGTAGGGTCCC | 62 °C |
| qPCR_Eef1a1 | ACAAGCGAACCATCGAAAAG | GTCTCGAATTTCCACAGGGA | 59°C |
| qPCR_Dchs1 | GGCCTGCCTCCTTTAGTCTC | TGTCAGCATCTGTGGCTGTT | 59°C |
| **name** | **gRNA#1** | **gRNA#2** |  |
| chr11:80480578-80480766 | TATTCTTGTTGCTTTTACAG | AAGAATACATCAACCCTTCA |  |
| chr2:35628229-35628358 | GAAATGGAAAAGCATAGTGA | AGGAATTTAAGGAGTCCCAC |  |
| chr13:97246240-97246462 | CACAACCAACAAATGGACCA | CTGGTTTCCCAGCACAGCTG |  |
| chr10:121029536-121029699 | ATTGGCTGAGCAAGGCAGAG | CTGTCAGTCTGTCCTCGAGT |  |
| chr7:105777824-105778152 | TTCATGTGGACTGACACAGC | CCCTATTCTCCTTTCTTTCC |  |
| offtarget (lacZ) Kalebic et al. 2016 | TGCGAATACGCCCACGCGAT | - |  |
