# Supplementary material for: Assessment and site-specific manipulation of DNA (hydroxy-)methylation during mouse corticogenesis
Source: Life Sci Alliance. 2019 Feb 27;2(2):e201900331. doi: 10.26508/lsa.201900331 (PMC6394126; doi:10.26508/lsa.201900331)
Supplement: Supplementary file 4 [file LSA-2019-00331_TableS2.doc]

Table S2. Antibodies used in this study

| name | cat. number | company | dilution |
| --- | --- | --- | --- |
| Tbr2 | ab23345 | Abcam | 1:500^1^ |
| BrdU | ab6326 | Abcam | 1:500^2^ |
| GFP | 600-101-215 | Rockland | 1:500 |
| Cy3 anti rabbit | 711-165-152 | Jackson Immunoresearch | 1:750 |
| Cy5 anti rabbit | 711-175-152 | Jackson Immunoresearch | 1:750 |
| Cy3 anti rat | 712-165-153 | Jackson Immunoresearch | 1:750 |
| Alexa-488 anti goat | 711-545-152 | Jackson Immunoresearch | 1:750 |

^1^Requires antigen retrieval (60 min at 70°C 0.01 M sodium citrate pH 6).

^2^Requires antigen retrieval (25 min at 37°C 2 M HCL).
